# Nucleoredoxin 1 in Wheat: Genomic Analysis and Demonstration of Its Role in Redox Homeostasis and Stress Resilience

**DOI:** 10.1002/pld3.70130

**Published:** 2025-12-09

**Authors:** Muhammad Sajawal Ghafoor, Rabia Naz, Adil Hussain, Asia Nosheen, Humaira Yasmin, Muhammad Sajjad, Wayne Thomas Shier, Rumana Keyani

**Affiliations:** ^1^ Department of Biosciences COMSATS University Islamabad Pakistan; ^2^ Department of Medicinal Chemistry, College of Pharmacy University of Minnesota Minneapolis Minnesota USA; ^3^ Department of Agriculture Abdul Wali Khan University Mardan Khyber Pakhtunkhwa Pakistan; ^4^ School of Biosciences Cardiff University Cardiff UK

**Keywords:** CRISPR‐Cas9, isoprenoid biosynthesis, MEP pathway, Nucleoredoxin 1 (NRX1), reactive oxygen species (ROS), redox homeostasis, stress tolerance, thioredoxin domain

## Abstract

Nucleoredoxin 1 (NRX1), a member of the redoxin superfamily, plays a critical role in maintaining redox homeostasis and enhancing stress tolerance in plants. We employed integrated in silico analyses and CRISPR‐Cas9‐based genome editing to functionally characterize NRX1 in *Triticum aestivum* (wheat) responding to salinity and infection by *Puccinia striiformis*. We identified five NRX1 proteins coded by three homeologs, with each containing conserved thioredoxin‐like domains and a Cys‐rich C‐terminal region. Sequence analysis predicted cytosolic and chloroplast localization, and promoter analysis predicted interaction with numerous *cis*‐regulatory elements responsive to stress and hormones, including ABRE, MeJARE, and LTRE motifs. Expression profiling revealed significant upregulation of *NRX1* in response to both salinity and *P. striiformis* infection. Protein–protein interaction analysis via STRING predicted strong co‐expression of NRX1 with 4‐hydroxy‐3‐methylbut‐2‐enyl diphosphate reductase (HDR) and thioredoxins, implicating NRX1 in regulating the methylerythritol phosphate pathway—crucial for isoprenoid biosynthesis and reactive oxygen species detoxification. CRISPR‐Cas9‐mediated knockout lines *nrx1‐b* and *nrx1‐bd* showed increased susceptibility of mutant plants to salinity and stripe rust infection. The total chlorophyll content was significantly reduced, and higher accumulation of malondialdehyde and decreased activities of catalase, superoxide dismutase, peroxidase, and ascorbate peroxidase were recorded compared to wild type (BW208) wheat. These results indicate NRX1 is an important regulator of redox signaling and stress adaptation in wheat, likely functioning through modulation of antioxidant enzymes and isoprenoid pathway intermediates. This study provides mechanistic insights into wheat stress biology and highlights NRX1 as a valuable molecular target for developing stress‐resilient wheat cultivars under climate change scenarios.

## 1 Introduction

Wheat (*Triticum aestivum L*.) is one of the most important cereal crops globally, serving as a staple food for nearly 40% of the world's population (K. Sharma and Sharma 2025). It is cultivated on over 218 million hectares worldwide, making a significant contribution to global food security (Statista, n.d.). However, wheat productivity is frequently challenged by a range of abiotic (drought, heat, and salinity) and biotic (pathogens and pests) stresses. These stressors disrupt cellular homeostasis, primarily by inducing the excessive accumulation of reactive oxygen species (ROS), which leads to oxidative damage to cellular components, including proteins, lipids, and nucleic acids. To mitigate oxidative stress, plants have evolved intricate antioxidant defense systems, comprising both enzymatic and non‐enzymatic mechanisms (P. Sharma et al. 2012). Key enzymatic antioxidants include catalase (CAT), superoxide dismutase (SOD), peroxidases (POD), and ascorbate peroxidase (APX), which collectively scavenge ROS to maintain cellular redox balance. Within this framework, the redoxin superfamily—encompassing thioredoxins, glutaredoxins, and nucleoredoxins—plays a central role in modulating redox signaling, detoxifying ROS, and regulating the activity of antioxidant enzymes (Zandi and Schnug 2022). Among these, Nucleoredoxin 1 (NRX1) is a redox‐active oxidoreductase characterized by the presence of three thioredoxin domains and one C1‐like domain (Marchal et al. 2014). In *Arabidopsis thaliana* , NRX1 has been shown to modulate hydrogen peroxide (H_2_O_2_) scavenging pathways and influence plant sensitivity to oxidative stress (Kneeshaw et al. 2017). However, the functional characterization of NRX1 in wheat, particularly its potential crosstalk with key metabolic pathways such as the methylerythritol phosphate (MEP) pathway that governs isoprenoid biosynthesis and contributes to ROS detoxification, remains largely unexplored.

In this study, we approached the problem of understanding the role of NRX1 in regulating redox homeostasis and stress resilience in wheat by conducting an extensive bioinformatic analysis of it in the wheat genome, and testing the results by preparing *NRX1* gene knockout wheat lines and comparing their responses to biotic and abiotic stress with those of wild‐type wheat. This study uses an alternate method to provide confirmation of the observations of Zhang et al. (2021) that disrupted functioning of *NRX1* in wheat causes increased susceptibility to an abiotic stress. The present study also demonstrates that *NRX1* genes enhance resistance in wheat to two additional, previously unstudied stress types. Drought and salt stress, although both osmotic, trigger markedly distinct redox and transcriptional signatures (Sewelam et al. 2020; Zandalinas and Mittler 2022). Salinization is an important abiotic stress agent in many parts of the world due to rising sea levels and extended irrigation with poor‐quality water. Fungal infection is an important biotic stress type for wheat cultivation in northern latitudes and higher elevations. In Pakistan, for example, salinization is an important stressor in irrigated wheat in the Punjab and Sindh, while stripe rust (*Puccinia striiformis*) infection is an important stressor to wheat grown in Himalayan regions.

## 2 Results

The role of NRX1 in regulating redox homeostasis and stress resilience in wheat was investigated by initially conducting a comprehensive bioinformatics analysis of the wheat genome to identify and characterize wheat *NRX1* homologs with respect to number and structure, predicted subcellular localization, and control by promoter *cis*‐elements in abiotic and biotic stress. This analysis and its predictions were then evaluated by creating *NRX1* gene knockout lines of wheat using CRISPR‐Cas9‐based genome editing techniques and characterizing them with respect to phenotypic and biochemical responses, notably those involved in redox homeostasis, to abiotic (salinity) and biotic (infection by *P. striiformis*) stresses in comparison with the corresponding responses by wild‐type wheat.

### 2.1 Identification and Structural Characterization of NRX1 in Wheat

BLASTP analysis using the *A. thaliana* NRX1 protein as a query identified three putative homologous genes in the wheat ( *T. aestivum* ) genome, located on chromosomes 2A, 2B, and 2D (Table 1). These genes encode five distinct protein isoforms: NRX1.1a, NRX1.1b, NRX1.2a, NRX1.3a, and NRX1.13b. All predicted wheat NRX1 proteins exhibited high sequence similarity to the Arabidopsis NRX1 and contained conserved thioredoxin domains, indicating a potential redox‐regulatory role. Domain validation through SMART and PFAM confirmed the presence of three redoxin domains and a C1‐like domain in each isoform, consistent with their classification within the thioredoxin superfamily (Figure S1). Physicochemical properties calculated using the Expasy ProtParam tool (Swiss Institute of Bioinformatics, n.d.) revealed that the molecular weights of wheat NRX1 proteins ranged from approximately 63.37 to 65.12 kDa. Their theoretical isoelectric points (pI) were between 4.78 and 4.91, indicating a slightly acidic nature (Table 1). These values suggest potential solubility and stability in diverse cellular environments. Subcellular localization predictions indicated that these proteins predominantly localize in the cytoplasm. However, additional signals suggest distribution to the chloroplast, nucleus, endoplasmic reticulum, vacuole, cytoskeleton, and extracellular space (Table 1), pointing toward multifunctional roles in various cellular compartments.

**TABLE 1:** Structural and biochemical features of wheat Nucleoredoxin 1 isoforms.

Transcript ID	Name	Chr no.	Gene length	Gene location	Transcript length	CDS	Protein length	Protein wt. (kDa)	pI	Predicted localization
Traes_2AL_D80447132.1	NRX1.1a	2A	4330	2245..6574	2115	1731	576	63.701	4.82	cyto: 8, extr: 3, nucl: 1, cysk: 1, ER/vacu: 1
Traes_2AL_D80447132.2	NRX1.1b	2A	2749	3685..6433	1755	1755	584	65.122	4.91	cyto: 8, chlo: 5, plas: 1
Traes_2BL_34819D129.1	NRX1.2a	2B	4125	4083..8207	1743	1743	580	63.803	4.78	cyto: 6, extr: 5, chlo: 2, nucl: 1
Traes_2DL_28DFAC79D.1	NRX1.3a	2D	2762	3921..6682	1755	1719	572	63.372	4.86	cyto: 8, chlo: 2, cysk: 2, nucl: 1, ER/vacu: 1
Traes_2DL_28DFAC79D.2	NRX1.3b	2D	4331	2587..6917	2162	1734	577	63.681	4.84	cyto: 6, extr: 5, nucl: 1, cysk: 1, ER/vacu: 1

### 2.2 Phylogenetic Analysis and Conserved Motif Distribution

**FIGURE 1:**
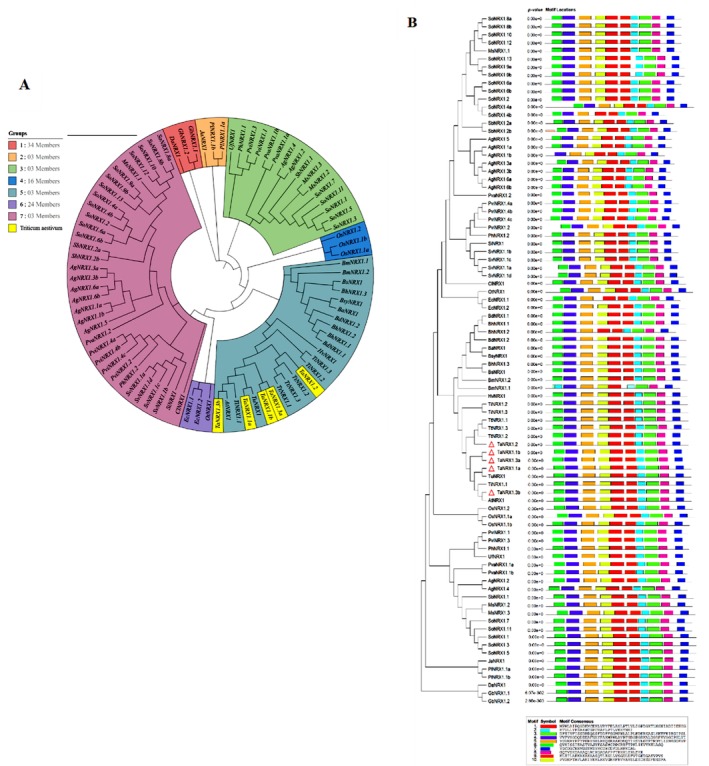
Phylogenetic analysis of evolutionary relationships among NRX1 genes in 30 monocot species, identifying seven clades or groups of species believed to have evolved from a common ancestor. (A) Wheat NRX1 genes (yellow) clustered with NRX1s from *Hordeum vulgare* , *T. turgidum* , *A. tauschii* , *T. intermedium* , and *T. Urartu* . (B) Conserved motifs among the NRX1 proteins were identified using the MEME Suite, with parameters set to detect up to 10 motifs ranging from 6 to 50 amino acids in width. Motifs are color‐coded revealing that similar motifs were present in all the proteins.

**FIGURE 2:**
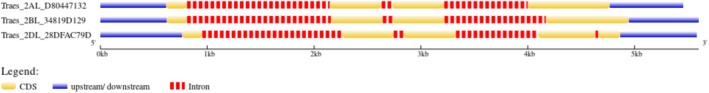
Structure and exon‐intron distribution in *NRX1* homeologs in wheat ( *Triticum aestivum* )*.*

Phylogenetic analysis was conducted on genes for NRX1 proteins from *T. aestivum* and 29 other plant species. The analysis revealed clustering into seven distinct clades, reflecting evolutionary relationships among the 30 species (Figure 1). Wheat *NRX1*s grouped closely with orthologs from *Hordeum vulgare* , *Triticum turgidum* , *Aegilops tauschii* , *Thinopyrum intermedium* , and *Triticum urartu* , indicating strong conservation within the Triticeae lineage. Among all the 30 species, *Saccharum officinarum* had the highest number of genes for NRX1 proteins (17), followed by *Andropogon gerardi* (9), *Panicum virgatum* (6), and *T. aestivum* (5). Other species, such as *Setaria viridis* (4), *Miscanthus sinensis* , *Oryza sativa* , and *Sorghum bicolor* (3 each), displayed variable NRX1 gene counts, suggesting lineage‐specific gene expansion (Figure 1A). MEME analysis identified 10 conserved motifs across all wheat NRX1 protein genes, including characteristic redoxin catalytic sites, supporting the potential functional roles of the proteins in redox regulation (Figure 1B). Additionally, gene structure analysis using GSDS revealed conserved exon–intron patterns among wheat *NRX1* genes, highlighting the evolutionary stability of their gene architecture (Figure 2).

### 2.3 Subcellular Localization and Promoter Analysis

**FIGURE 3:**
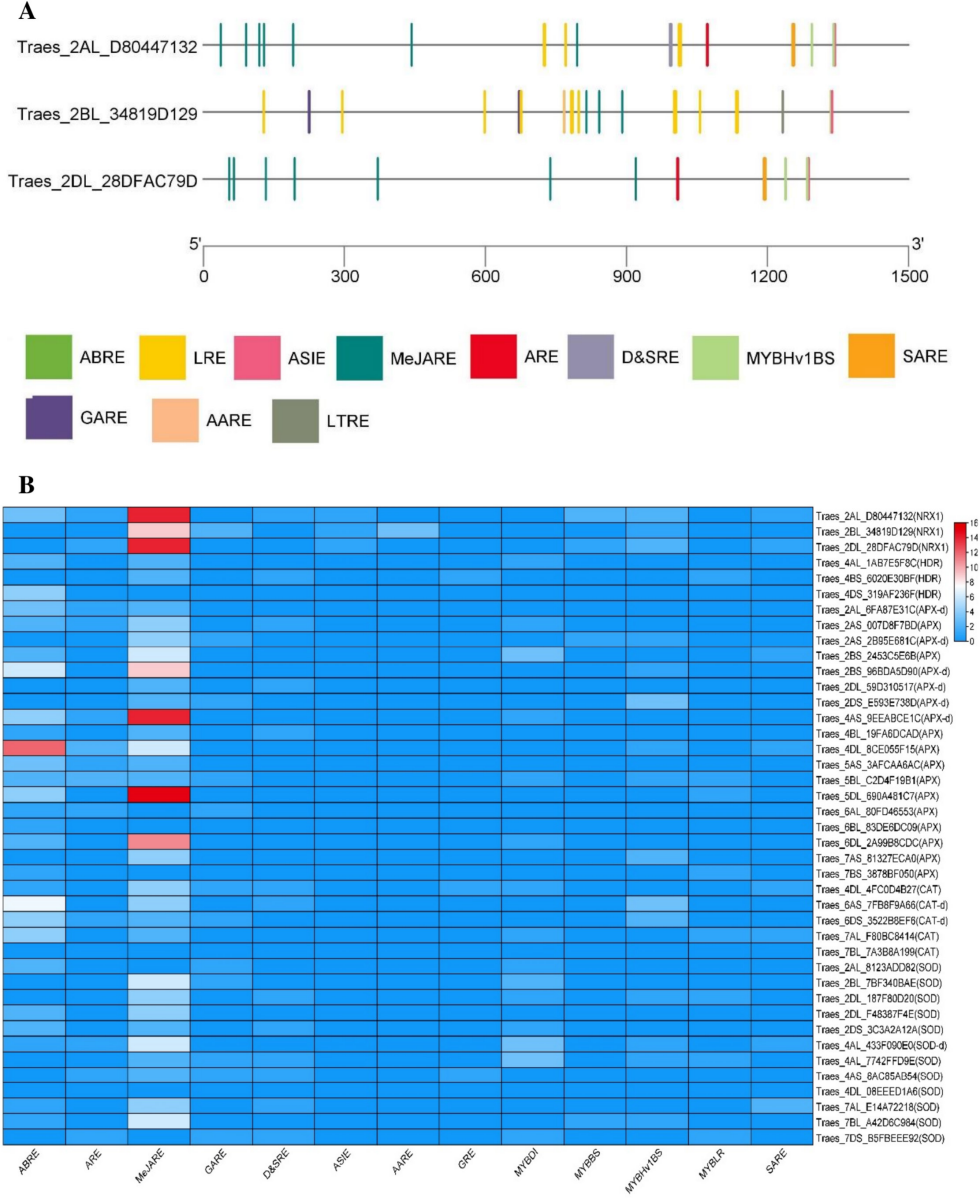
Promoter analysis of the *NRX1* gene of wheat using PlantCARE. (A) *cis*‐Regulatory elements of *NRX1*; (B) Comparative promoter analysis of *NRX1s*, antioxidant genes (*catalases, SODs, APXs*), and *HDR* genes.

Subcellular localization prediction using WoLF PSORT indicated that all five wheat NRX1 proteins are primarily localized in the cytosol, aligning with their proposed roles in redox regulation. NRX1.1a and NRX1.2a also showed potential extracellular localisation, while NRX1.1b, NRX1.2a, and NRX1.3a exhibited the possibility of chloroplast localization, suggesting additional roles in plastid redox processes (Table 1). Promoter analysis using PlantCARE revealed a total of 79 *cis*‐regulatory elements (CREs) across the *NRX1* gene promoters, encompassing both hormone‐responsive and stress‐related elements (Figure 3A,B). Hormonal regulatory motifs included MeJA‐responsive elements, ABRE (abscisic acid), ERE (ethylene), GARE (gibberellin), SARE (salicylic acid), and ARE (auxin), suggesting complex hormonal crosstalk in NRX1 regulation. Stress‐responsive elements included ASIE (anoxic stress), D&SRE (defense and stress), and LTRE (low‐temperature responsiveness), highlighting their potential roles under environmental challenges. Notably, multiple MYB binding sites (MYBBS) involved in flavonoid biosynthesis, along with five MYBHv1 binding sites (MYBHv1BS), were also detected, indicating transcriptional regulation via MYB transcription factors. Comparative promoter analysis of related antioxidant genes (catalases, SODs, APXs) and *HDR* genes revealed that while most showed a diverse regulatory element profile, *HDR* had fewer *cis*‐elements, suggesting a more constitutive or housekeeping expression pattern (Figure 3B).

### 2.4 Post‐Translational Modification (PTM) Prediction

**FIGURE 4:**
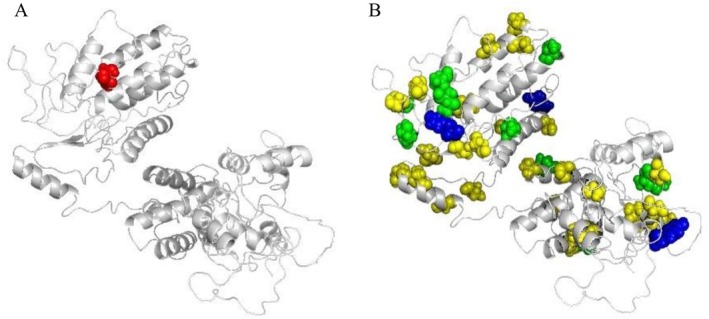
Prediction of glycosylation and phosphorylation sites in 3D secondary structure of the TaNRX1 protein. (A) The glycosylation site is highlighted in red. (B) Phosphorylation sites are highlighted (serine is yellow, threonine is green, and tyrosine is blue).

PTM prediction using NetPhos (DTU Health Tech Bioinformatic Services, n.d.‐a) identified multiple serine and threonine phosphorylation sites across all NRX1 proteins, suggesting involvement in kinase‐mediated signaling (Figure 4B). NetNGlyc analysis (DTU Health Tech Bioinformatic Services, n.d.‐b) predicted one N‐glycosylation site in each of NRX1s (Figure 4A), indicating potential for post‐translational modulation of NRX1 stability and function. The secondary structure of all these proteins was predicted using the SOPMA webserver, and *α*–helices, *β*–sheets, extended strands, and random coils were predicted between 35.42%–36.71%, 10.66%–11.03%, 22.41%–23.78%, and 29.37%–31.03%, respectively (Figure S2). Disordered regions were analyzed using the Mobi DB server (https://mobidb.bio.unipd.it). All three proteins showed no disordered regions and contained the functional domain (Figure S3).

### 2.5 Protein–Protein Interaction and Pathway Enrichment Analysis

**FIGURE 5:**
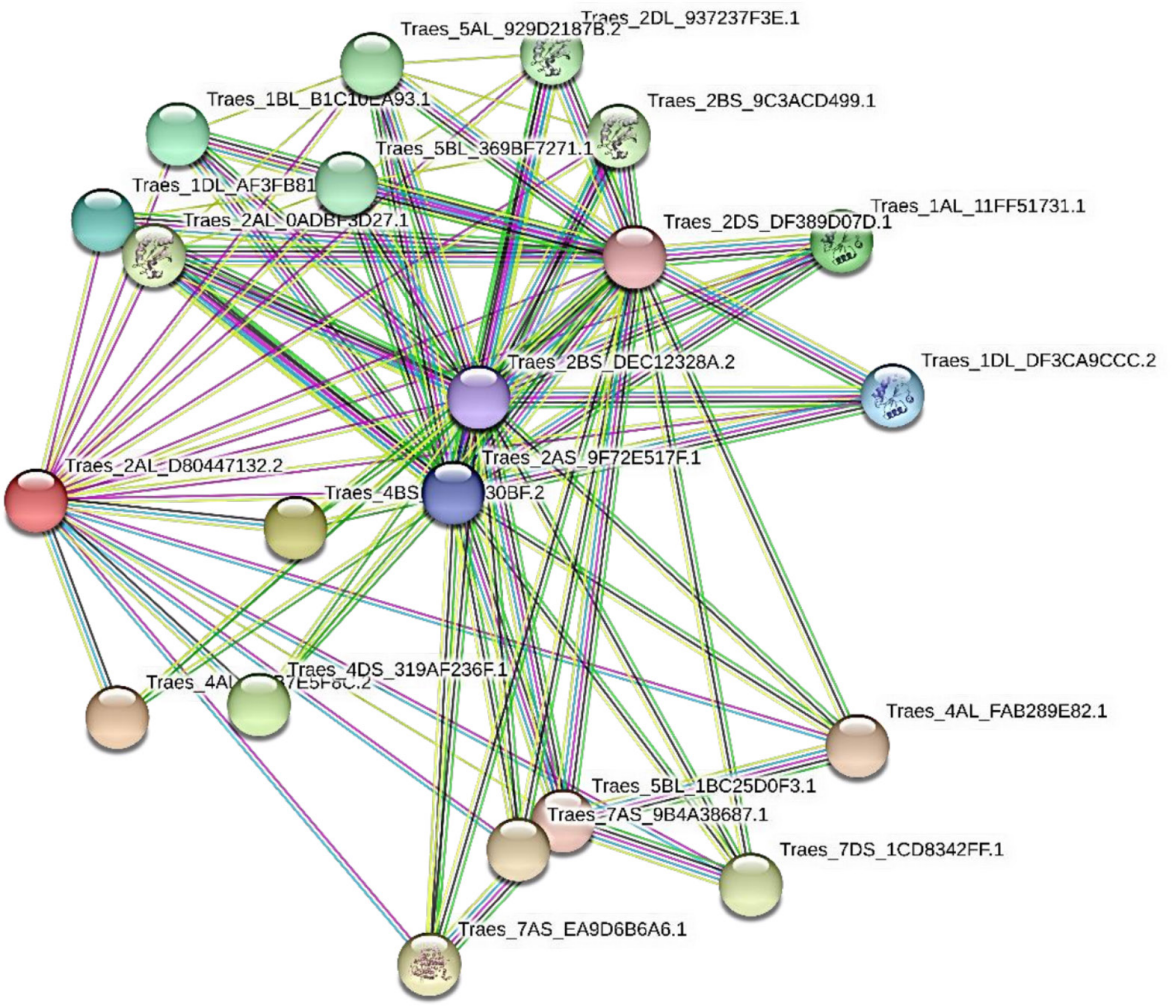
Protein–protein interaction network of wheat Nucleoredoxin 1 (NRX1) with other identified proteins in wheat.

Protein–protein interaction analysis using the STRING database predicted that wheat NRX1 proteins are highly connected with other redox‐related proteins, including thioredoxins, peroxiredoxins, and redoxins, consistent with them playing a central role in redox regulation networks (Figure 5). A particularly strong interaction was predicted with 4‐hydroxy‐3‐methylbut‐2‐enyl diphosphate reductase (HDR), a key enzyme in the methylerythritol phosphate (MEP) pathway, which links NRX1 to isoprenoid biosynthesis. Table 2 below summarizes functional annotations and subcellular localization of NRX1‐interacting proteins. Many of these proteins localize to chloroplasts, mitochondria, cytoplasm, and nuclei—compartments critical for redox homeostasis (Table 2). For instance, several thioredoxins and thioredoxin‐like proteins (e.g., Traes_2BL_9C3ACD499.1, Traes_2DL_397237F3E.1, and Traes_5BL_369BF7271.1) co‐localize in the chloroplast and mitochondria, indicating potential coordination of redox signaling across organelles. KEGG pathway enrichment further indicated NRX1's association with antioxidant defense and metabolic pathways, especially isoprenoid biosynthesis via the MEP pathway. These findings collectively suggest that wheat NRX1 proteins may act as critical regulators of redox‐dependent signaling and secondary metabolism.

**TABLE 2:** Functional descriptions and predicted subcellular localizations of proteins able to interact with NRX1.

Protein ID	Functions	Subcellular localization
Traes_7DS_1CD8342FF.1	PEROXISOMAL ACYL‐COENZYME A OXIDASE 2	Mito: 5, Chlo: 4, Cyto: 3, Nucl: 2
Traes_4AL_1AB7E5F8C.2	4‐hydroxy‐3‐methylbut‐2‐enyl diphosphate reductase/HMBPP reductase	Chlo: 10.5, Chlo_mito: 7.5, Mito: 3.5
Traes_1DL_DF3CA9CCC.2	THIOREDOXIN	Cyto: 10, Pero: 3, Cysk_nucl: 1
Traes_4BS_6020E30BF.2	4‐hydroxy‐3‐methylbut‐2‐enyl diphosphate reductase/HMBPP reductase	Chlo: 12, Mito: 2
Traes_2BS_9C3ACD499.1	THIOREDOXIN H1	Cyto: 14
Traes_2AL_0ADBF3D27.1	THIOREDOXIN H1	Cyto: 12, Chlo: 2
Traes_5BL_1BC25D0F3.1	DNAJ HOMOLOG SUBFAMILY C MEMBER	Chlo: 10, Nucl: 3, Mito: 1
Traes_7AS_9B4A38687.1	Acyl‐CoA oxidase (ACOX)	Pero: 9, Cyto: 3.5, Cyto_nucl: 2.5, Golg: 1
Traes_2DL_937237F3E.1	THIOREDOXIN H1	Cyto: 12, Chlo: 2
Traes_1BL_B1C10EA93.1	Thioredoxin (Thioredoxin_9)	Chlo: 5, Cyto: 5, Extr: 2, Cysk: 1.5, Cysk_nucl: 1.5
Traes_1DL_AF3FB8142.1	Thioredoxin (Thioredoxin_9)	Cyto: 9, Chlo: 2, Cysk: 1.5, Cysk_nucl: 1.5, Pero: 1
Traes_5BL_369BF7271.1	THIOREDOXIN‐LIKE PROTEIN HCF164, CHLOROPLASTIC	Chlo: 14
Traes_4DS_319AF236F.1	4‐hydroxy‐3‐methylbut‐2‐enyl diphosphate reductase/HMBPP reductase	Chlo: 10, Mito: 4
Traes_2BS_DEC12328A.2	Pyridine nucleotide‐disulphide oxidoreductase (Pyr_redox) //Thioredoxin (Thioredoxin)	Cysk: 7.5, Cysk_plas: 4.5, Nucl: 4, Cyto: 2
Traes_4AL_FAB289E82.1	PEROXISOMAL ACYL‐COENZYME A OXIDASE 2	Cyto: 9, Chlo: 2, Nucl: 2, Plas: 1
Traes_7AS_EA9D6B6A6.1	Isovaleryl‐CoA dehydrogenase	Chlo: 5.5, Chlo_mito: 5.5, Mito: 4.5, Cyto: 3, Cysk: 1
Traes_5AL_929D2187B.2	THIOREDOXIN‐LIKE PROTEIN HCF164, CHLOROPLASTIC	Nucl: 5, Cysk: 4, Chlo: 3, Extr: 2
Traes_2AS_9F72E517F.1	NADPH‐DEPENDENT THIOREDOXIN REDUCTASE 3	Cyto: 7, Nucl: 3, Cysk: 3, Golg: 1

### 2.6 Genome‐Wide Expression Profiling of *NRX1* and Antioxidant Genes

**FIGURE 6:**
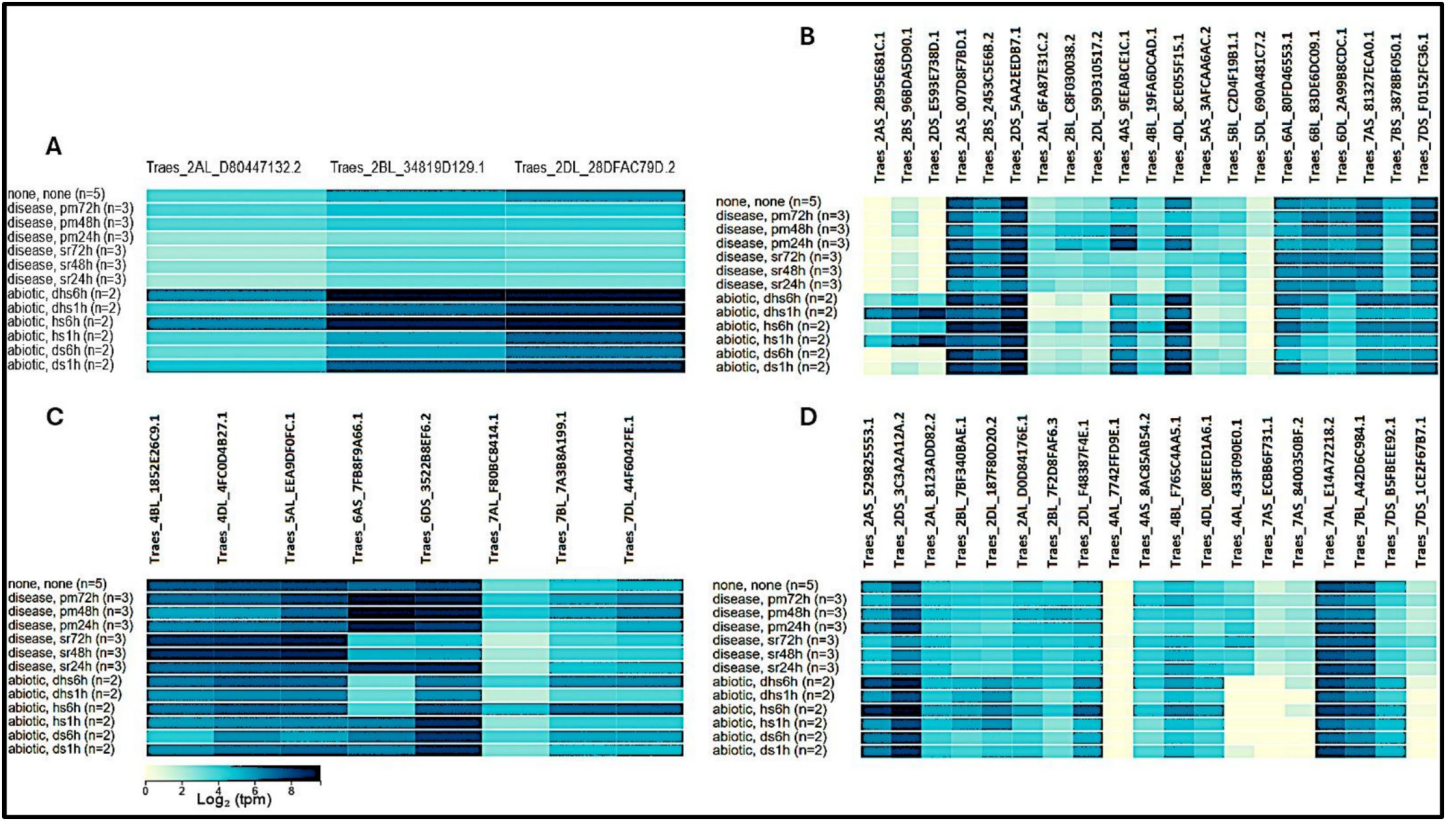
Change in genome‐wide expression of (A) *NRX1* and antioxidant enzymes including (B) ascorbate peroxidase, *APX*; (C) *catalase*; and (D) *superoxide dismutase, SOD* under biotic (infection by powdery mildew and stripe rust) and abiotic (heat and drought) stresses.

Expression data retrieved from WheatExp (www.wheat‐expression.com) revealed that most wheat *NRX1* genes are substantially upregulated in response to both abiotic and biotic stresses (Figure 6). Under drought conditions, *NRX1* expression showed a transient induction followed by suppression, consistent with a time‐dependent regulatory role during stress adaptation (Figure 6A). Similarly, biotic stresses such as powdery mildew and stripe rust also triggered *NRX1* expression, suggesting its involvement in plant defense signaling (Figure 6A). In parallel, key antioxidant defense genes—including *superoxide dismutase (SOD), catalase (CAT)*, and *ascorbate peroxidase (APX)*—also displayed dynamic expression patterns under the same stress conditions. *SOD* genes were notably responsive to drought and stripe rust (Figure 6D), while *CAT* genes showed variable upregulation under both heat and biotic stress conditions (Figure 6C). *APX* genes exhibited strong stress‐inducible expression under all tested conditions, particularly under heat stress (Figure 6B). These coordinated expression trends suggest that *NRX1* may function in a broader redox regulatory network, interacting with core antioxidant systems to mitigate oxidative damage during environmental stress.

### 2.7 CRISPR‐Cas9 Editing of *NRX1* Genes

**FIGURE 7:**
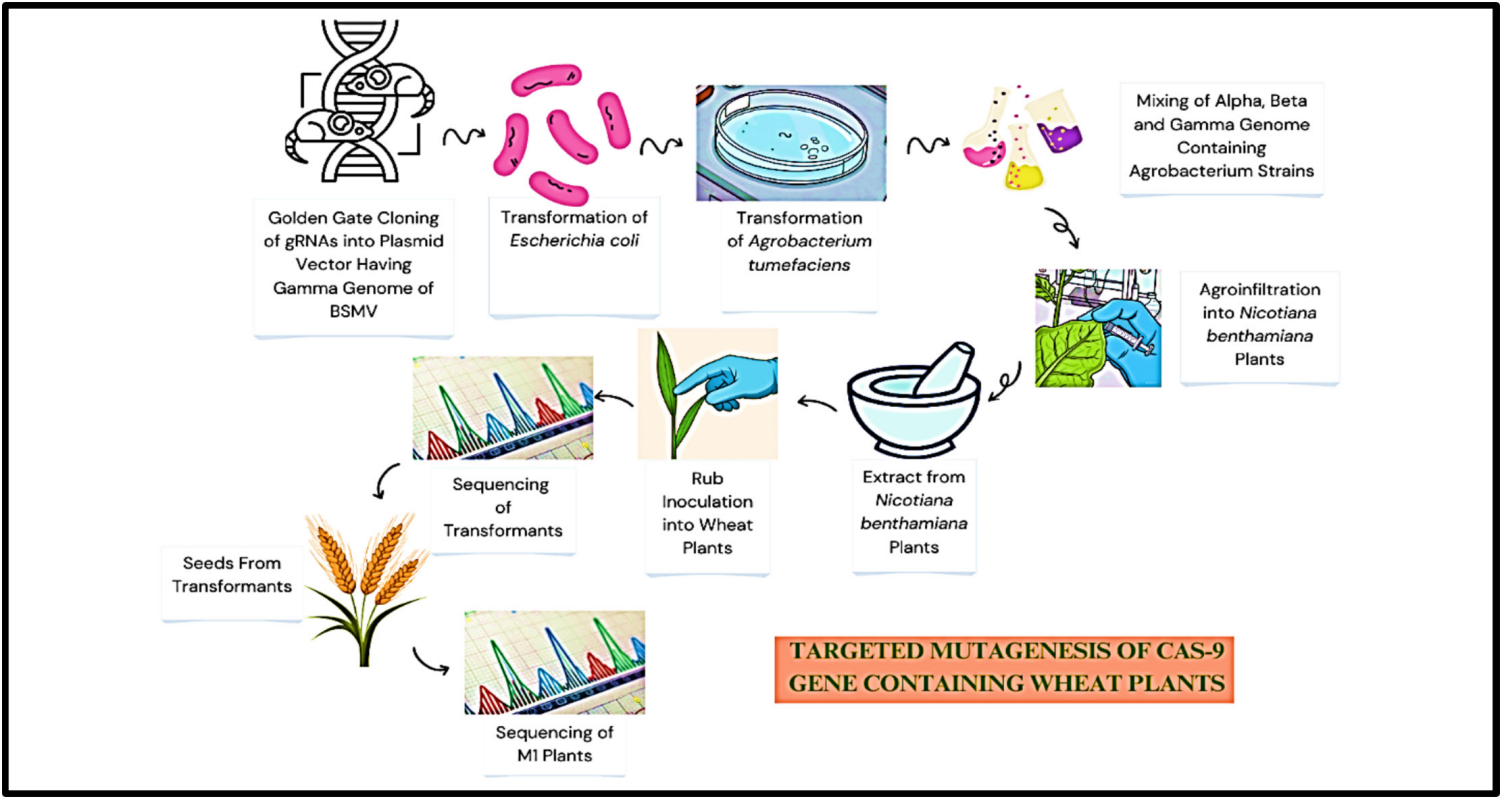
Flow chart of experiments for the development of *NRX1* knockout mutant wheat plants. Each of the three guide RNAs synthesized with suitable overhangs was assembled using golden gate cloning (Marillonnet and Grützner 2020), transformed into *Escherichia coli* and *Agrobacterium tumefaciens* (Hu et al. 2019), which were used to infect the leaves of *Nicotiana benthamiana* (Yuan et al. 2011). Ground leaf extract was used to rub‐inoculate the leaves of the BW208 wheat line expressing the Cas 9 gene (Li et al. 2021; Smedley et al. 2021; Wang et al. 2022).

**FIGURE 8:**
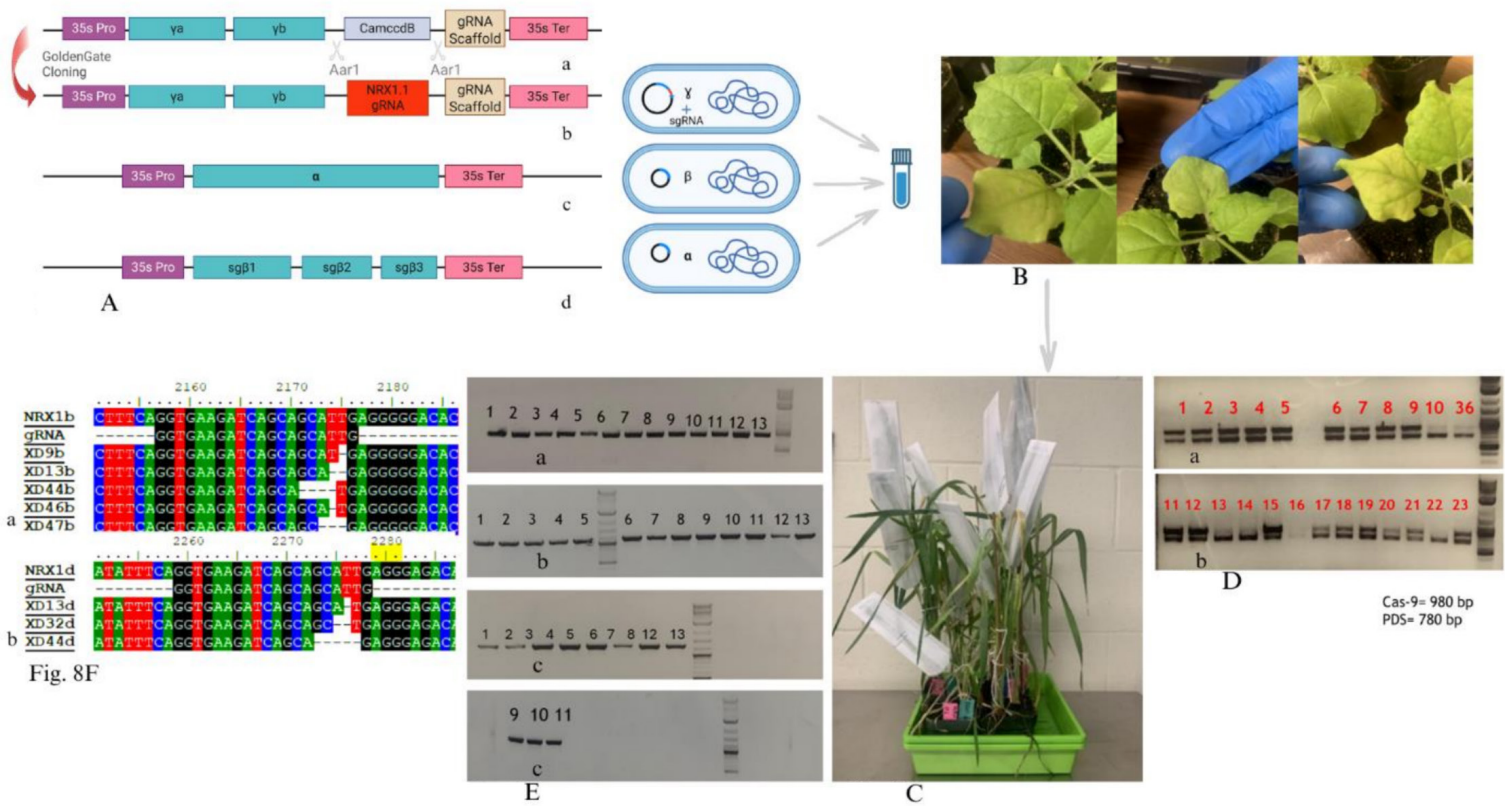
Steps in the preparation of *NRX1* knockout mutant wheat plants. (A) Preparation of vector with gamma genome of BSMV virus, before and after the cloning of gRNA. Alpha and Beta parts of the BSMV genome are shown. (B) BSMV‐infected *Nicotiana benthamiana* plants. (C) Cas9‐Wheat plants infected with BSMV virus. (D) Results for the PCR amplification of the *Cas9* gene and *PDS* gene for confirmation of *Cas9* presence. (E) PCR amplification of the target regions from a, b, and d sub‐genomes of inoculated wheat plants. (F) Mutations in the target regions.

Exonic regions of NRX1 were targeted in Cas9‐wheat plants using BSMV‐delivered gRNAs (Figure 7). Targeted wheat plants exhibited mutations confirmed by PCR amplification and sequencing of the target regions (Figure 8E,F). Genome‐specific primers were designed for the amplification of target regions of *NRX1* alleles from A (Figure 8E‐a), B (Figure 8E‐b), and D (Figure 8E‐c) genomes of inoculated plants to evaluate the mutation rates induced by different BSMV vectors. Target regions were amplified using the genomic DNA from the M0 and M1 generations. We identified three plants with deletions in the B and D subgenomes. Seeds from the confirmed mutants in the M1 generation were used for downstream phenotypic and biochemical assays in the M2 generation.

### 2.8 Phenotypic Analysis Under Stress Conditions

**FIGURE 9:**
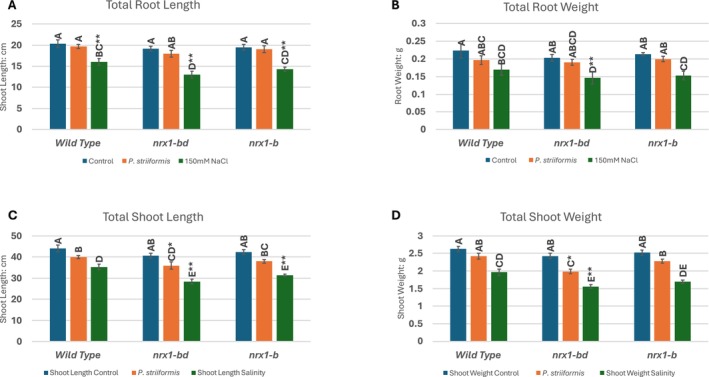
Effects of mutations in *nrx1‐bd* and *nrx1‐b* wheat plant lines on (A) root length, (B) root weight, (C) shoot length, and (D) shoot weight 10 days after the application of 150‐mM salt stress and stripe rust infection.

To evaluate the physiological impact of CRISPR‐Cas9‐mediated mutations in *NRX1*, two mutant lines *nrx1‐bd* and *nrx1‐b* were used (labeled as XD44 and XD13 in Figure 8F and Table 3). *Nrx1‐bd* and *nrx1‐b* contain small deletions in the conserved active sites, as shown in Figures 8F and S4 and Table S1. They result in frameshift mutations in the modified NRX1 protein produced, rather than knockout of the complete *NRX1* gene, which would be a desirable additional experiment. Phenotypic traits were assessed in mutants (*nrx1‐bd* and nrx*1‐b*) and wild‐type BW208 (Bobwhite 208) wheat lines under stripe rust and salinity stress (Figure 9). Under control conditions, shoot lengths ranged between 42–44 cm and root lengths between 18 and 21 cm across all lines. Under salinity stress, *nrx1‐bd* and *nrx1‐b* exhibited reduced shoot and root lengths (43.5% and 35% in *nrx1‐bd* and 47.4% and 36% in *nrx1‐b*, respectively), compared to BW208 (24.5% shoot; 27% root). Fresh biomass of shoots and roots also declined sharply in mutants, with a reduction of 55.6% and 49% in shoot weight and 38.6% and 39% in root weight in *nrx1‐bd* and *nrx1‐b*, respectively, under salinity, compared to BW208's 33% and 31% reductions, respectively. These results highlight compromised stress tolerance in mutant lines. Stripe rust infection caused major reductions in shoot weight. There was a 22% and 10.6% reduction in shoot weight in *nrx1‐bd* and *nrx1‐b*, respectively, compared to an 8.4% reduction in the shoot weight of BW208.

**TABLE 3:** Number of mutants in M1 generation.

M0 Plant‐ID	M1 progeny	Mutants
XD9	10	5
XD13	7	2
XD32	12	0
XD44	8	4
XD46	10	3

Stripe rust infection was evaluated based on infection type and severity of symptoms. Wild type BW208 exhibited the lowest infection type, with an average score of 2 on a 0–9 scale, which indicates a relatively resistant response, while *nrx1‐b* and *nrx1‐bd* plants showed higher infection types of 4 and 5, respectively. Disease severity, based on the percentage of damaged leaf area, showed a similar trend. BW208 showed low average severity at 16%, while the mutants *nrx1‐bd* and *nrx1‐b* exhibited increased severities of 26% and 29%, respectively. The results suggest the susceptibility of BW208 toward stripe rust increased after mutation in the *NRX1* genes.

The altered sensitivity observed in *nrx1‐bd* and *nrx1‐b* mutants is consistent with a functional role of NRX1 in redox regulation, stress resilience, and potentially isoprenoid‐mediated antioxidant defense pathways. The summarized phenotypic data are presented in Figure 9.

### 2.9 Chlorophyll Content Analysis Under Stress Conditions

**FIGURE 10:**
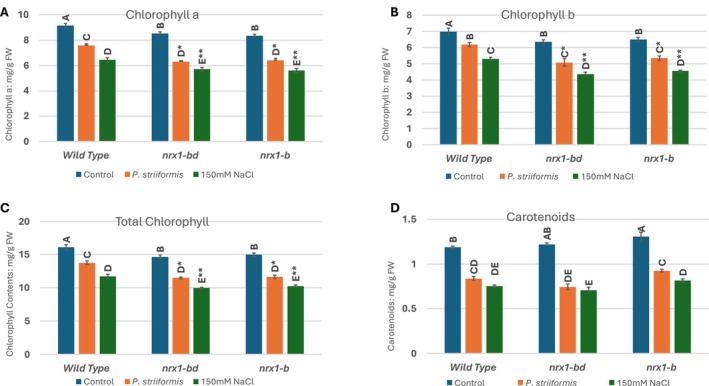
Effects of mutations on (A) chlorophyll a, (B) chlorophyll b, (C) total chlorophyll, and (D) carotenoids contents of *nrx1‐bd* and *nrx1‐b* wheat plant lines 10 days after the application of 150‐mM salt stress and stripe rust infection.

Total chlorophyll content was measured to assess the impact of salinity and stripe rust stress on the photosynthetic efficiency of CRISPR‐Cas9‐induced mutant lines (*nrx1‐bd* and *nrx1‐b*) compared to the wild‐type (BW208). Chlorophyll was extracted using the dimethyl sulfoxide (DMSO) method, and absorbance was recorded at 470 nm, 645 nm, and 665 nm for total chlorophyll calculation. Under control conditions, all lines showed high chlorophyll content, with BW208 and *nrx1‐b* exhibiting slightly higher levels, while *nrx1‐bd* had slightly lower content (Figure 10). Chlorophyll contents were significantly affected by salinity in all the plants (*p* < 0.05). Stripe rust also had a significant effect on normal plants, but the chlorophyll contents of the mutants were affected more (*p* < 0.05). The highest reduction in chlorophyll contents was observed in *nrx1‐bd* mutants under stress conditions. A reduction of up to 35% in chlorophyll a, 24% in chlorophyll b, and 28% in total chlorophyll content was observed in the mutants under stripe rust stress compared to 20%, 13%, and 17% reduction in the normal BW208 plants under stripe rust. Salinity caused significantly more reduction in chlorophyll contents with 49% in chlorophyll a, 45% in chlorophyll b, and 47% reduction in total chlorophyll, while the reduction was 42%, 32%, and 37% respectively, in normal BW208 plants under salinity stress (*p* < 0.05). In summary, there was a major impact on the carotenoid contents of the mutants under stress conditions. Overall, the photosynthetic pigments in *NXR1* knockout plants were substantially more impacted by both kinds of stresses compared to wild‐type plants (Figure 10).

### 2.10 Oxidative Stress Markers and Antioxidant Enzyme Activity in Wheat Plant Lines Under Stress Conditions

**FIGURE 11:**
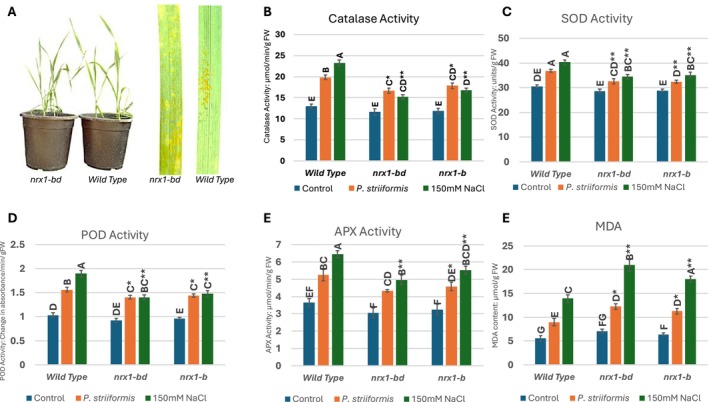
Effects of NRX1 mutations on antioxidant enzyme activity and MDA content of *nrx1‐bd* and *nrx1‐b* wheat plant lines 10 days after the application of 150 mM salt stress and stripe rust infection. (A) Mutants were impacted by stripe rust and salinity substantially more than wild type (BW280). The effect of the mutations was compared to wild type for antioxidant enzyme activity content of (B) catalase, (C) superoxide dismutase (SOD), (D) peroxidase (POD), (E) ascorbate peroxidase (APX), and (F) malondialdehyde (MDA) in wheat plant lines under stress.

To further investigate the physiological effects of the CRISPR‐Cas9‐induced mutations in *NRX1*, oxidative stress levels and antioxidant enzyme activities were evaluated under control, salinity, and stripe rust conditions. To investigate the redox‐regulatory role of *NRX1* under stress conditions, lipid peroxidation and antioxidant enzyme activity in wild‐type (BW208) and CRISPR‐Cas9 mutant wheat lines (*nrx1‐bd* and *nrx1‐b*) exposed to salinity and stripe rust were evaluated. The results highlight clear differences in oxidative stress indicators between wild‐type and mutant genotypes (Figure 11). Malondialdehyde (MDA) levels, a marker of lipid peroxidation and membrane damage, remained low under control conditions across all genotypes.

An increase in the MDA content was observed in plants under stress conditions, and MDA contents of mutants were affected significantly more (*p* < 0.05, Tukey's HSD test), indicating heightened membrane damage due to insufficient ROS detoxification. The increase in MDA content was higher in the case of salinity compared to that of stripe rust. The increase in MDA content with the onset of stripe rust stress was up to 43% in the mutants compared to 41% wild‐type plants. Similarly, the mutants showed an increase of MDA up to 66% under salinity stress compared to a 61% increase in normal plants under salinity stress. This shows that mutants had more ROS, and they had lost their ability to deal with their lipid peroxidation under stress conditions. Although the MDA contents were higher, there was not much difference in MDA contents of *nrx1‐bd* and *nrx1‐b* type plants under stripe rust.

The activity of key antioxidant enzymes followed a similar trend. Superoxide dismutase (SOD) activity in BW208 remained consistently high under both stress conditions, effectively dismutating superoxide radicals. However, SOD activity declined in *nrx1‐bd* and *nrx1‐b*, suggesting a reduced capacity to counteract oxidative stress at the primary level. The increase in SOD activity under stripe rust stress was up to 12% in the mutants when compared to 17% in BW208 plants. Similarly, the mutants showed an increase of almost 18% under salinity stress compared to a 25% increase in normal plants under salinity stress.

Similarly, an increase in peroxidase (POD) and catalase (CAT) and ascorbate peroxidase (APX) activities was significantly lower in the mutant lines compared to wild type plants. The increase in POD activity under stripe rust stress was up to 23% in the mutants when compared to 33% in wild type plants, which is almost 10% less increase. Similarly, the mutants showed an increase of up to 34% under salinity stress compared to a 45% increase in normal plants under salinity stress.

A little less increase in catalase activity under stripe rust stress was observed in the mutants, compared to the increase of 35% in BW208 plants, while APX activity also showed a similar kind of change. Similarly, the mutants showed up to 30% increase in catalase activity under salinity stress compared to a 43% increase in activity in normal plants under salinity stress and APX activity increased up to 40% in mutants compared to a 42% increase in wild‐type plants under salinity stress, reinforcing the overall trend of weakened antioxidant defense in the edited lines. Collectively, these biochemical findings provide strong evidence that NRX1 disruption impairs oxidative stress tolerance, as evidenced by higher MDA accumulation and reduced antioxidant enzyme activity in mutant plants. The wild‐type BW208 line's superior performance under stress conditions further validates NRX1's essential role in redox regulation and stress resilience in wheat. In summary, antioxidant enzyme activities were reduced in *NRX1* knockout mutants with the largest effect on catalase while MDA levels were increased (Figure 11).

### 2.11 qPCR Expression Analysis

**FIGURE 12:**
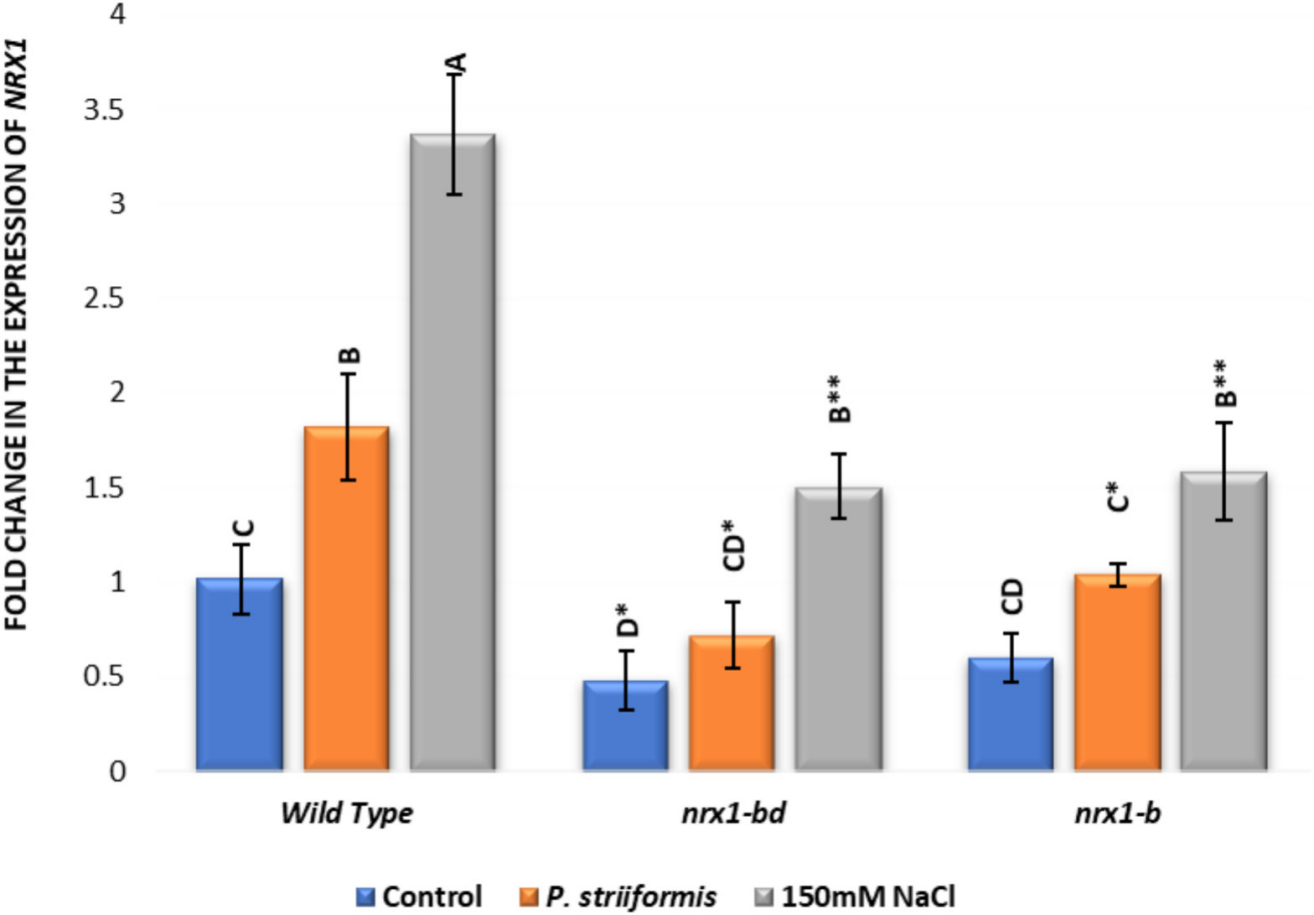
qPCR analysis of *NRX1* gene expression in knockout BW280 wheat lines *nrx1‐bd* and *nrx1‐b*, compared to wild‐type plants following treatment with a biotic stress (*P. striiformis* stripe rust disease) and abiotic stress (salinity due to 150‐mM NaCl).

qPCR expression analysis showed a reduced expression of *NRX1* genes in the mutants *nrx1‐bd* and *nrx1‐b*, compared to wild‐type plants under unstressed conditions as well as following abiotic salinity stress and biotic stress in the form of stripe rust disease (Figure 12). An increase in expression of *NRX1* was observed under both types of stress. The expression was higher under salinity compared to stripe rust infection. Reduced expression of *NRX1* genes in the mutants was associated with the increased susceptibility of wheat plants to the effects of both salinity and stripe rust. We observed an increase in expression of *NRX1* genes under stress in mutants also, but the increase was lower than the increase in NRX1 expression in normal plants under stress. Expression was more affected in *nrx1*‐bd than in *nrx1*‐b.

## 3 Discussion

NRX1, found in all eukaryotes, is a thioredoxin‐like protein. It controls different cellular processes by controlling the activity of different proteins through thiol‐disulfide exchange reactions (Funato and Miki 2007; Gupta et al. 2024). It plays an important role in redox regulation and maintains the redox state of the cell. It was named thioredoxin because of the presence of thioredoxin domains and its presence in the nucleus, although subcellular fractionation of NIH3T3 mouse fibroblast cells found it to be a predominantly cytosolic protein (Funato et al. 2006).

We identified gene sequences for five NRX1 proteins in wheat, coded by three genes. Sequence analysis indicated that they are predominantly localized in the cytosol and chloroplast. The role of NRX1 in the regulation of cytokinins and hence leaf senescence has already been reported in rice (Yao et al. 2023). While analyzing the protein–protein interactions using the STRING database, we found that most of the interacting proteins are thioredoxins or are involved in the biosynthesis of secondary metabolites or fatty acid beta‐oxidation. Subcellular localization of these interacting proteins indicated that most of these proteins are targeted to the cytoplasm and the chloroplast. This shows that NRX1 acts not only for the protection of antioxidant enzymes but also for the regulation of other regulatory biomolecules.

Reduced activity of antioxidant enzymes has already been reported under oxidative stress in knockout mutants of the *NRX1* gene. We observed in this study that mutations in the *NRX1* gene made wheat plants susceptible to salinity stress. This mutation caused a 7% lower increase in SOD activity, an 11% lower increase in POD activity, and a 13% lower increase in catalase activity when compared to the increase in activity of these antioxidants in wild type BW208 plants under similar salinity stress. We observed up to a 5% greater increase in MDA contents in the mutants, while there was a 7%, a 13%, and a 10% greater loss of chlorophyll a, chlorophyll b, and total chlorophyll contents, respectively, in BW208 plants under similar salinity stress.

Our results showed that mutation in the *NRX1* gene was also associated with an increase in susceptibility of wheat plants toward stripe rust, which we measured as a significant (*p* < 0.05, Tukey's HSD test) decline in photosynthetic pigments, decreased activity of antioxidant enzymes, and a higher increase in MDA contents compared to wild type plants. This result is consistent with the disrupted functioning of *NRX1* causing a reduction in the activity of antioxidant enzymes and making plants susceptible to salinity stress, as reported earlier for plants under some but not all other kinds of abiotic stresses (Cha et al. 2022; Liu et al. 2025; Zhang et al. 2021). Li et al. (2016) reported that *NRX1* gene silencing in cotton resulted in reduced wilt resistance to *V. dahliae* infection due to higher apoplastic concentration of ROS. Similarly, Gauthier et al. (2014) reported that salicylic acid‐induced expression of *NRX1* improved resistance to downy mildew in grapes. On the other hand, enhanced resistance against *Pseudomonas syringae* and *Alternaria brassicola* has been reported in CRISPR/Cas9 mutants of the *NRX1* gene of tomato (Cha et al. 2023). To understand the underlying mechanism, we evaluated protein–protein interactions and expression of the interacting proteins. These results predicted that activity levels of the oxygen‐sensitive protein 4‐hydroxy‐3‐methylbut‐2‐enyl diphosphate reductase (HDR), also known as lysis‐tolerant B (LytB) or isoprenoid synthesis H (IspH), would be closely linked to *NRX1*. HDR catalyzes the last step of the MEP pathway in bacteria and all plants. It is an iron–sulfur protein and Cys residues involved in iron–sulfur cluster formation are present in the HDR of all photosynthetic organisms. The MEP pathway is compartmentalized in the plastids. Synthesis of dimethylallyl diphosphate (DMAPP) and isopentenyl diphosphate (IPP) is catalyzed by HDR in the last step of the MEP pathway. Monoterpenes, isoprene, phytol, tocopherols, plastoquinones, carotenoids, and plant hormones abscisic acid and gibberellin are synthesized in plastids from components produced by the MEP pathway. The activity and expression of *HDR* have been assessed in different plant species. Light intensity has been reported to cause an overexpression of *HDR* and other MEP pathway genes in *Arabidopsis thaliana* (Pokhilko et al. 2015). This increase is part of the oxidative stress response of the plant because higher light intensity will lead to enhanced production of isoprenoids, which play a role in dealing with oxidative stress. *HDR* and *NRX1* showed similar expression patterns under biotic and abiotic stress conditions, and they have a similar subcellular location. The catalytic site of HDR[4Fe‐4S] is sensitive to the redox state of the cell (Misra et al. 2024). We propose that NRX1 acts to maintain the catalytic site of HDR[4Fe‐4S] in the active form even under a changing redox status in the cell under stress conditions. HDR determines the IPP/DMAPP ratio in plants and influences the stress responses of plants by changing isoprenoid balance (Krause et al. 2020). IPP is used by plants for the synthesis of volatile terpenes, which act as defense compounds, and it serves as a precursor for making cytokinin and gibberellins. On the other hand, DMAPP is used for making carotenoids, salicylic acid (SA), and membrane sterols, which play roles in plant stress resistance by different mechanisms (Rodríguez‐Concepción 2010).

A change in the enzymatic activity of HDR can affect the IPP/DMAPP ratio in plants. Reduced activity of HDR results in lower DMAPP concentration, which results in reduced synthesis of salicylic acid and impaired salicylic acid‐mediated defense against biotrophic pathogen infection (Abreu and Munné‐Bosch 2009).

Under abiotic stress, reduced activity of HDR will lead to reduced synthesis of carotenoids, which will result in ROS accumulation and oxidative damage to plants (Zahra et al. 2021). HDR has conserved cysteine residues, which are susceptible to oxidative damage under oxidative stress (Perez‐Gil et al. 2024). NRX1 maintains the HDR catalytic site through its thioredoxin activity. Hence, an altered NRX1 activity will affect the activity of HDR, thereby changing the way the plant responds to oxidative stress.

Defense mechanisms against biotrophs are salicylic acid‐dependent (Mishra et al. 2024), while defense mechanisms against necrotrophs are jasmonic acid and ethylene‐dependent (Glazebrook 2005). This difference explains why altering the level of NRX1 activity can increase resistance to some forms of plant stress while reducing it to others. It has already been reported that the disrupted functioning of HDR will result in a reduced amount of SA, which will result in increased activity by jasmonic acid and ethylene pathways (SA‐JA antagonism) (Misra et al. 2024) and also that *NRX1* disruption will cause increased JA (SA suppression) and an increase in phytoalexins (Mata‐Pérez and Spoel 2019). Thus, plants with a mutation reducing the *NRX1* gene activity were better able to defend themselves against necrotrophic pathogens, but they were more susceptible to infection by biotrophic pathogens, such as *P. striiformis* in wheat. Increased susceptibility of NRX1 mutants of wheat toward stripe rust can also be attributed to less DMAPP in mutants, which results in reduced synthesis of antimicrobial terpenoids.

The integration of in silico and CRISPR‐based approaches in this study has revealed NRX1's multifaceted role in regulating the plant responses to stress. Its interaction with HDR suggests a link between redox homeostasis and isoprenoid biosynthesis. Targeted mutagenesis studies validated computational predictions about NRX1's actions and interactions, providing a framework for future genomic studies in wheat.

## 4 Conclusions

By establishing a mechanistic link between NRX1 and redox regulation, the MEP pathway, and antioxidant defense, our findings offer novel insights into wheat stress biology. The identification of the central role of NRX1 in redox homeostasis not only enhances our understanding of plant stress adaptation in wheat, but also positions NRX1 as a promising molecular target for breeding or engineering wheat varieties with improved resilience to oxidative and environmental stress. This work contributes to the broader goal of achieving sustainable crop production under changing climate conditions. These results indicate that NRX1 is a promising target for genetic improvement of crops under climate stress. Future work should focus on the regulation of expression of NRX1 and complementation studies to further validate its function.

## 5 Materials and Methods

### 5.1 Sequence Retrieval and Characterization of Wheat *NRX1* Genes

To identify *NRX1* homologs in wheat ( *T. aestivum* ), the protein sequence of *A. thaliana NRX1* was retrieved from The Arabidopsis Information Resource (TAIR), and the domain sequences (three thioredoxins and one C1‐like domain) were used as a query for a BLASTP search against the wheat genome (IWGSC RefSeq v2.2) through the database Phytozome (https://phytozome‐next.jgi.doe.gov/). A stringent e‐value threshold of < 1e−5 was used to ensure specificity. The candidate wheat *NRX1* sequences were screened for the presence of thioredoxin‐like domains using the PFAM and SMART databases, confirming domain conservation and structure. Basic physicochemical properties such as isoelectric point (pI), molecular weight, and protein length were determined using the ProtParam tool available on the Expasy server (Swiss Institute of Bioinformatics n.d.).

### 5.2 Phylogenetic Analysis, Gene Structure, and Motif Identification

Multiple sequence alignment of NRX1 protein sequences from wheat and 29 other plant species ( *S. officinarum* , *A. gerardi* , *P. virgatum* , *T. aestivum* , *S. viridis* , *Brachypodium hybridum*, *Miscanthus sinensis* , *Paspalum vaginatum* , *Sorghum bicolor* , *Oryza sativa* , *T. intermedium* , *T. turgidum* , *Brachypodium distachyon* , *Brachypodium mexicanum* , *Eleusine coracana* , *Gossypium barbadense* , *Panicum hallii* , *Pharus latifolius* , *Brachypodium arbuscula*, *Brachypodium stacei*, *Brachypodium sylvaticum* , *Chasmanthium laxum* , *Dioscorea alata* , *H. vulgare* , *Joinvillea ascendens* , *Oropetium thomaeum*, *Setaria italica* , *Urochloa fusca* , *A. tauschii* , *T. urartu* ) was conducted using MEGA‐X software (Kumar et al. 2018). The phylogenetic relationships were inferred using the Neighbor–Joining (NJ) method with 1000 bootstrap replications to assess the reliability of the clades. Gene structure analysis was performed using the Gene Structure Display Server (GSDS 2.0) (Hu et al. 2015), where exon‐intron arrangements were visualized by comparing genomic DNA and coding DNA sequences (CDS). Conserved motifs among the NRX1 proteins were identified using the MEME Suite (v5.4.1), with parameters set to detect up to 10 motifs ranging from 6 to 50 amino acids in width (Bailey et al. 2009).

### 5.3 Subcellular Localization Prediction and Promoter Analysis

The subcellular localization of wheat NRX1 proteins was predicted using the WoLF PSORT web server (Horton et al. 2007) to infer the likely compartmentalzation within the cell, such as cytosol, chloroplast, nucleus, or mitochondria. To understand the regulatory potential of *NRX1* genes, *HDR* genes, and genes for antioxidant enzymes, promoter regions extending 1.5 kb upstream of the start codon were obtained from the wheat genome using the database Phytozome (https://phytozome‐next.jgi.doe.gov/). These promoter sequences were submitted to the PlantCARE database (Lescot et al. 2002) for the identification of CREs.

### 5.4 Prediction of Post‐Translational Modifications (PTMs)

Post‐translational modifications play a crucial role in regulating protein function, stability, and interactions. Phosphorylation sites on serine, threonine, and tyrosine residues were predicted using the NetPhos 3.1 server (DTU Health Tech Bioinformatic Services, n.d.‐a), providing both generic and kinase‐specific predictions. These phosphorylation events are essential for activating signal transduction pathways in response to stress. Glycosylation potential was evaluated using NetNGlyc 1.0 (DTU Health Tech Bioinformatic Services, n.d.‐b), where sequences were analyzed for the presence of N‐linked glycosylation motifs. A threshold score of 0.5 or higher, with a jury agreement of 9/9, was used to confirm high‐confidence glycosylation sites. These PTM predictions helped infer the potential post‐translational regulation of NRX1 proteins during stress responses. Secondary structures were predicted using the SOPMA web server (Geourjon and Deléage 1995). Disordered regions were analyzed using the Mobi DB server (Piovesan et al. 2021).

### 5.5 Protein–Protein Interaction and Gene Expression Profiling

To explore the functional interactions of NRX1, protein–protein interaction networks were constructed using the STRING database (www.string‐db.org). Amino acid sequences of three wheat NRX1 proteins were analyzed via the STRING database, and the network was filtered using an enrichment *p* value threshold to include the most significant interactors. Special attention was given to proteins co‐expressed and co‐localized with NRX1, such as HMBPP reductase (HDR) and thioredoxins, suggesting links with redox regulation and the methylerythritol phosphate (MEP) pathway. Additionally, in silico expression profiles of NRX1 and major antioxidant genes (e.g., SOD, CAT, and APX) were analyzed using the WheatExp database (Borrill et al. 2016). Genes of different antioxidant enzymes were identified using already published data, specifically for SOD genes (Jiang et al. 2019), catalases (Zhang et al. 2022), and APXs (Tyagi et al. 2020).

### 5.6 CRISPR‐Cas9‐Mediated Editing of *NRX1* Genes

To functionally validate the role of NRX1, CRISPR‐Cas9‐mediated genome editing was employed. Guide RNAs (gRNAs) (Table 4) were designed to target conserved exonic regions of *NRX1s* using the CRISPR‐direct online tool (www.crispr.dbcls.jp). Three gRNAs were designed from the conserved regions in *NRX1* homologs. Each gRNA was synthesized with overhangs suitable for Golden Gate cloning into a modified Barley Stripe Mosaic Virus (BSMV)‐based vector, pGY036, which contains the gamma part of the BSMV genome. Golden Gate cloning was performed using standard protocols (Marillonnet and Grützner 2020), followed by transformation into *Escherichia coli* and *Agrobacterium tumefaciens* for vector propagation and Agro‐infiltration (Yuan et al. 2011). Agrobacteria were also transformed separately with plasmid constructs having the alpha and beta genomes of the BSMV virus. At this stage, we had three kinds of Agrobacterium strains, each containing one of the three parts (alpha, beta, and gamma with gRNA) of the BSMV genome. An equal concentration of all three strains was mixed and infiltrated into the underside of approximately the 5th true leaf of *Nicotiana benthamiana* plants using a 1‐mL needleless syringe (Hu et al. 2019). For the mechanical inoculation of wheat plants, we harvested *A. tumefaciens* ‐infiltrated leaves of *Nicotiana benthamiana* after 10 days of infiltration. The leaves were finely ground in 20 mM Na‐phosphate buffer (pH 7.2) containing 1% silicon carbide powder, and the sap was rub‐inoculated onto the plants of “Bobwhite 208” BW208 wheat line already expressing the Cas9 gene for the delivery of gRNA via viral expression (Li et al. 2021; Smedley et al. 2021; Wang et al. 2022). Editing efficiency was evaluated using PCR amplification and Sanger sequencing of target regions from both systemically infected tissues and progeny plants. The Voytas laboratory, University of Minnesota, provided plant materials and vectors for these experiments, and the mutants were developed there (www.voytaslab.umn.edu).

**TABLE 4:** Three gRNAs were designed from different exonic regions from three conserved regions in all three homeologs of the *NRX1* genes.

	Strand	gRNA with PAM	Location in the cDNA
NRX1	+	5′‐GGTGAAGATCAGCAGCATTGAGG‐3′	92–114
	+	5′‐CCCCTTCAATGCAGAGAAGCTGG‐3′	941, 962–963, 984
	−	5′‐CCGCCATGCCAGCGGTTTACACC‐3′	177–199

### 5.7 Stress Application and Plant Responses

To assess the impact of *NRX1* gene editing, mutant and control wheat plants were grown under normal and stress conditions induced by salinity and stripe rust infection. For stripe rust infection, the urediniospore mixture was applied to wheat leaves with a paintbrush. Inoculated seedlings were placed under high humidity for 24 h at 10°C in the dark. After 24 h, inoculated leaves were placed under normal conditions with 75% relative humidity. Salinity stress was applied by irrigating plants with a 150 mM solution of NaCl. Morphological parameters, such as root and shoot lengths and fresh biomass, were recorded 10 days after the application of both stresses. Photosynthetic pigments, including chlorophyll a, b, and total chlorophyll, were measured from leaf tissues using the method described by Ronen and Galun (1984). Lipid peroxidation was assessed using the method described by Heath and Packer (1968). Superoxide dismutase (SOD), catalase (CAT), and peroxidase (POD), ascorbate peroxidase (APX) activities were measured as described by Demirezen Yilmaz and Uruç Parlak 2011; Taggar et al. 2012; Alavifard et al. 2012, and Altaf et al. 2022, respectively. The differences in physiological and biochemical responses between wild‐type and mutant lines helped validate the predicted role of NRX1 in modulating redox balance and stress tolerance.

### 5.8 qPCR Expression Analysis

qPCR expression analysis was done by using the conserved primers for all three homeologs of *NRX1* under stripe rust stress and salinity stress, 10 days after the application of stress. RNA extraction was performed using the Trizol method. RNA was resuspended in 30 μL of RNase‐free water. The RNA concentration was quantified using a NanoDrop spectrophotometer at 260 nm under RNase‐free conditions, and the RNA was stored at −80°C.

First‐strand cDNA synthesis was done by using the RevertAid First Strand cDNA Synthesis Kit (Thermo Fisher Scientific, Catalog#K1622). Maxima SYBR Green qPCR Master Mix (ThermoFisher Scientific) was used for real‐time expression analysis. The 12.5‐μL Maxima SYBR Green Master Mix (2×), 0.3‐μL forward primer, 0.3‐μL reverse primer, ≤ 500‐ng cDNA template, and nuclease‐free water were mixed to raise the volume to 25 μL. Reaction tubes were inserted into the cycler, and then the program for quantitative expression analysis on Mic qPCR (Bio‐Molecular System) was run. CT values were obtained for expression analysis.

### 5.9 Statistics

Experiments were conducted in a factorial design to assess the effects of both the stresses and mutations and their interactions on mutant plants compared to wild‐type plants. All experiments were performed with three replications, and data were collected and tabulated in Microsoft Excel. Means and standard deviations were calculated in Microsoft Excel. Statistical analyses were done by two‐way ANOVA followed by Tukey's HSD test at a 95% level of significance, and all pairwise means were compared.

## Author Contributions

Rumana Keyani, Adil Hussain, and Rabia Naz conceived and designed the study. Muhammad Sajawal Ghafoor performed the experiments. Data analysis was conducted by Muhammad Sajawal Ghafoor, Asia Nosheen, Humaira Yasmin, Muhammad Sajjad, and Rumana Keyani, and Wayne Thomas Shier provided laboratory facilities and technical guidance for cloning and CRISPR experiments. Wayne Thomas Shier and Rabia Naz drafted and revised the manuscript, with input from all authors.

## Funding

This research was funded in part by the Higher Education Commission of Pakistan, Government of Pakistan, Islamabad, Pakistan, under the International Research Support Initiative Program (IRSIP), HEC Indigenous Scholarship program, and International Foundation for Science (IFS).

## Conflicts of Interest

The authors declare no conflicts of interest.

## Supporting information


**Figure S1:** Functional domains in different NRX1 isoforms in wheat ( *Triticum aestivum* ). A: Traes_2AL_D80447132.1; B: Traes_2BL_34819D129.1; and C: Traes_2DL_28DFAC79D.1.
**Figure S2:** Secondary structures of Nucleoredoxin 1 Proteins: (A) Traes_2AL_D80447132.1, (B) Traes_2BL_34819D129.1, and (C) Traes_2DL_28DFAC79D.1.
**Figure S3:** Disordered region in Nucleoredoxin 1 proteins: (A) Traes_2AL_D80447132.1, (B) Traes_2BL_34819D129.1, and (C) Traes_2DL_28DFAC79D.1.
**Figure S4:** Multiple sequence alignment of five TaNRX1 proteins with NRX1 from *Arabidopsis thaliana* showing the active site sequences WCXPC in black blocks. Three thioredoxin sites in each TaNRX1 were observed and two of them had WCXPC active sites.
**Table S1:** Summary of observed DNA sequences in wheat knockout mutants selected for phenotypic analysis under stress conditions.

## Data Availability

The data that support the findings of this study are available from the corresponding author upon reasonable request.
