# Supplementary material for: Nucleoredoxin 1 in Wheat: Genomic Analysis and Demonstration of Its Role in Redox Homeostasis and Stress Resilience
Source: Plant Direct. 2025 Dec 9;9(12):e70130. doi: 10.1002/pld3.70130 (PMC12688404; doi:10.1002/pld3.70130)
Supplement: Supplementary file 1 — Figure S1: Functional domains in different NRX1 isoforms in wheat ( Triticum aestivum ). A: Traes_2AL_D80447132.1; B: Traes_2BL_34819D129.1; and C: Traes_2DL_28DFAC79D.1. Figure S2: Secondary structures of Nucleoredoxin 1 Proteins: (A) Traes_2AL_D80447132.1, (B) Traes_2BL_34819D129.1, and (C) Traes_2DL_28DFAC79D.1. Figure S3: Disordered region in Nucleoredoxin 1 proteins: (A) Traes_2AL_D80447132.1, (B) Traes_2BL_34819D129.1, and (C) Traes_2DL_28DFAC79D.1. Figure S4: Multiple sequence alignment of five TaNRX1 proteins with NRX1 from Arabidopsis thaliana showing the active site sequences WCXPC in black blocks. Three thioredoxin sites in each TaNRX1 were observed and two of them had WCXPC active sites. Table S1: Summary of observed DNA sequences in wheat knockout mutants selected for phenotypic analysis under stress conditions. [file PLD3-9-e70130-s001.docx]

*Plant Direct* Supporting Information

**Figure S1** Functional domains in different NRX1 isoforms in wheat (*Triticum aestivum*). A: Traes_2AL_D80447132.1; B: Traes_2BL_34819D129.1; and C: Traes_2DL_28DFAC79D.1.

**Figure S2** Secondary structures of Nucleoredoxin 1 Proteins: (A) Traes_2AL_D80447132.1, (B) Traes_2BL_34819D129.1, and (C) Traes_2DL_28DFAC79D.1.

**Figure S3** Disordered region in Nucleoredoxin 1 proteins: (A) Traes_2AL_D80447132.1, (B) Traes_2BL_34819D129.1, and (C) Traes_2DL_28DFAC79D.1.


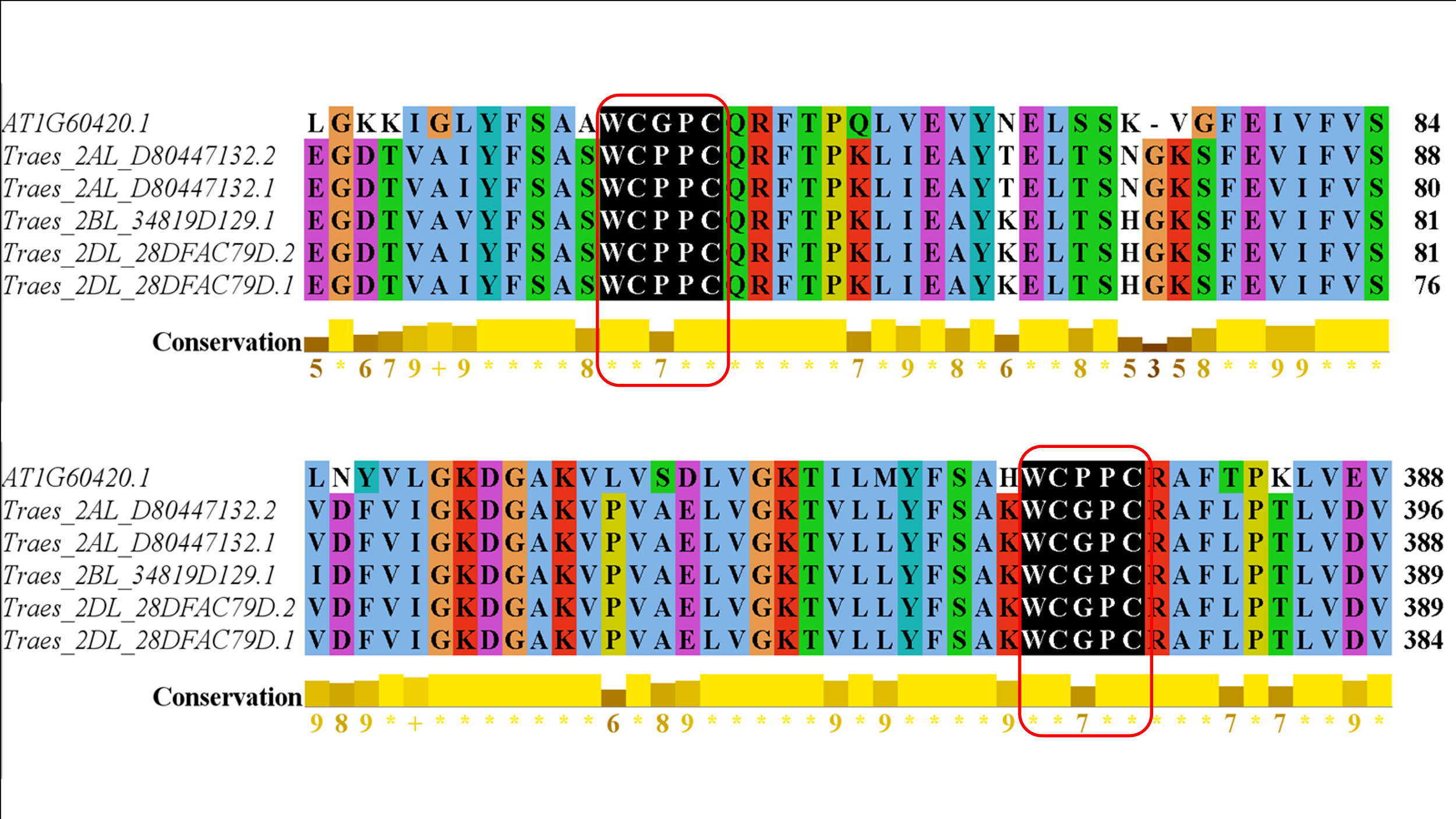


**Figure S4** Multiple sequence alignment of five TaNRX1 proteins with NRX1 from *Arabidopsis thaliana* showing the active site sequences WCXPC in black blocks. Three thioredoxin sites in each TaNRX1 were observed and two of them had WCXPC active sites.

**Table S1** Summary of observed DNA sequences in wheat knockout mutants selected for phenotypic analysis under stress conditions

partial sequence (2170-2177) partial sequence (2270-2279)

Wild type CAGCATTGA GCAGCATTG

*NRX1-bd* CA----TGA* GCA-----G*

*NRX1-b* CAGCA—GA* GCAGCA-TG*

*This deletion mutation will cause a frameshift mutation which will continue in the resulting NRX1 protein until a stop codon is created, resulting in truncation of the polypeptide chain.
